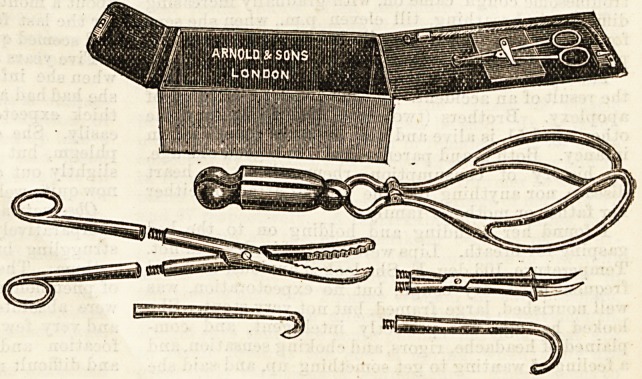# New Appliances and Things Medical

**Published:** 1894-01-13

**Authors:** 


### NEW APPLIANCES AND THINGS MEDICAL.

[All preparations, appliances, novelties, &c., of which a notice is desired, should be sent for the Editor, to care of The Manager, 428, Strand, London, W.C.]

### THE "COMPACTUM" MIDWIFERY CASE.

The above illustration shows a very portable Midwifery set, which contains the following: Simpson's long forceps (folding); Craniotomy forceps and perforator, to fit into the same handles; blunt hook and Crochet (jointed) Frænum scissors, and female catheter. All the instruments are nickel plated, and fit into a morocco leather case measuring 8½ by 3½ by 3½ in. The makers are Arnold and Sons, of West Smithfield, London.

## Figures and Tables

**Figure f1:**